# Type2 Diabetes Mellitus Remission After Sleeve Gastrectomy in Patients 60 Years and Older—A Multicenter Study

**DOI:** 10.3390/jcm15218613

**Published:** 2026-11-05

**Authors:** Pawel Jaworski, Mateusz Wityk, Piotr Major, Wiesław Tarnowski, Natalia Dowgiałło-Gornowicz

**Affiliations:** 1 Department of General, Oncological and Bariatric Surgery, Centre of Postgraduate Medical Education, Orłowski Hospital, 00-416 Warsaw, Poland; pawel.jaworski@cmkp.edu.pl (P.J.); tarnowskiwieslaw@me.com (W.T.); 2 Department of General and Oncological Surgery, Regional Health Centre, 59-300 Lubin, Poland; mateuszwityk@gmail.com; 3 Department of Medical Education, Jagiellonian University Medical College, 31-008 Cracow, Poland; majorpiotr@gmail.com; 4 Department of General, Minimally Invasive and Elderly Surgery, Collegium Medicum, University of Warmia and Mazury, 10-045 Olsztyn, Poland; natalia.dowgiallo@gmail.com

**Keywords:** older patients, bariatric surgery, metabolic surgery, type 2 diabetes mellitus

## Abstract

**Introduction**: Driven by impaired insulin secretion and insulin resistance, type 2 diabetes mellitus (T2DM) stands as a chronic metabolic disorder. While metabolic bariatric surgery (MBS) is highly effective, data on older populations remain limited. The objective of this study is to assess the long-term outcomes and clinical predictors of T2DM remission in patients aged 60 and older following sleeve gastrectomy. **Materials and Methods**: This retrospective cohort study evaluates data retrieved from the medical records of 129 patients aged ≥60 years with T2DM who underwent primary laparoscopic sleeve gastrectomy between 2008 and 2022. Baseline demographic characteristics, body mass index (BMI), T2DM duration, and treatment modalities were extracted. Metabolic outcomes, including total weight loss (%TWL) and excess weight loss (%EWL), were evaluated over a median follow-up of 6.0 years. **Results**: The cohort had a median age of 66 years, with females accounting for 62% of the population. At the final follow-up, the median %TWL was 19.4%, and the median %EWL was 47.8%. Overall, T2DM remission was achieved in 80 (62.0%) patients, whereas 16 (12.4%) patients experienced no clinical changes. Duration of T2DM of more than 10 years was associated with a lower likelihood of remission of T2DM (*p* = 0.007). **Conclusions**: Our findings demonstrate that a substantial proportion of patients aged 60 years and older achieved T2DM remission at the last available follow-up following sleeve gastrectomy. A decreased likelihood of remission correlated with longer preoperative T2DM duration, which highlights the clinical value of offering metabolic surgery early to eligible older patients.

## 1. Introduction

Characterized by sustained hyperglycemia, type 2 diabetes mellitus (T2DM) is a chronic metabolic disorder that stems from defective insulin secretion, deficient insulin action, or a combination of both factors [1]. Uncontrolled T2DM can give rise to a multitude of complications affecting microcirculation and macrocirculation, renal function, vision, as well as the nervous and immune systems [1]. Currently, the disorder impacts approximately one in ten individuals globally, with projections indicating a 25% increase in prevalence over the next two decades and a potential doubling of cases in developing nations. Notably, diabetes is responsible for more than 10% of total mortality, with over half of these fatalities occurring in individuals aged 60 and older [2,3,4].

T2DM is recognized as one of the most financially burdensome diseases. The direct costs incurred per individual may exceed $10,000 on an annual basis, while the indirect expenses are estimated to surpass $15,000 in developed nations [4]. The total annual global expenditure on diabetes is predominantly concentrated within the demographic aged 60 to 69, with costs exceeding $177 billion. Projections indicate that by the year 2030, global expenditure on the management of diabetes and its associated complications is anticipated to exceed $2 trillion [4,5,6].

For patients diagnosed with T2DM, metabolic bariatric surgery (MBS) represents a powerful tool to achieve both weight reduction and superior glycemic management. MBS not only yields substantial and sustained weight reduction but also engenders significant enhancements in insulin sensitivity and beta-cell functionality [7,8]. Many studies have elucidated that a considerable proportion of patients experience either partial or complete remission of T2DM post-surgery [7,8,9,10]. Furthermore, surgical intervention has been correlated with a diminished requirement for antidiabetic pharmacotherapy and a reduced incidence of complications associated with T2DM [10].

As the most frequently performed bariatric procedure worldwide, sleeve gastrectomy (SG) accounts for over 350,000 operations globally executed each year [11]. The enhancement and remission of T2DM after SG have been well-established and extensively documented for years. Nevertheless, information regarding the effect of SG on T2DM management in elderly patients is still limited and lacking [11,12,13,14,15,16].

This study examines how sleeve gastrectomy (SG) affects T2DM outcomes in patients aged 60 years and older.

## 2. Materials and Methods

### 2.1. Study Design and Population

This multicenter, retrospective study analyzed a comprehensive database of patients aged 60 and older who underwent sleeve gastrectomy (SG) in Poland between 2008 and 2022. Eligible participants met standard metabolic bariatric surgery (MBS) criteria, were aged ≥60 years, and had a confirmed diagnosis of T2DM. Patients with incomplete or inconsistent records were excluded from the analysis. The study contained data from two multicenter projects conducted in Poland on outcomes of MBS in patients over 65 years of age and in patients with a 10-year follow-up (BARI-10-POL). The two multicenter cohorts involved 11 bariatric centers. Each center included only patients who underwent their primary surgery and subsequent follow-up at that institution, thereby excluding duplicate records across centers. For additional comparative analysis, patients aged <60 years with T2DM who underwent SG and fulfilled the same procedural criteria were included as a younger comparator group. This comparison was considered exploratory because of differences in follow-up duration and baseline characteristics between the cohorts.

The database contained demographic characteristics of patients (sex, age, weight before surgery, body mass index (BMI) and information on T2DM (type of treatment, duration of T2DM) and hypertension. It also included information on the surgery (duration of surgery, length of hospital stay) and complications. The analysis is in line with STROBE guidelines. All reported outcomes correspond to the final follow-up available for each patient. The analysis was restricted to patients with complete data regarding postoperative type 2 diabetes remission at this specific follow-up. A clear distinction was made between the completeness of primary outcome data and the completeness of baseline covariates. Patients were included in the final analysis only if they had definitive T2DM status data at their final follow-up; missing baseline covariates (such as T2DM duration) were managed by reporting the exact denominator for each specific sub-analysis.

### 2.2. Surgical Technique and Outcomes

SG was performed according to the standard technique using a bougie size of 36F, starting 4–6 cm from the pylorus. Outcomes of MBS were described in accordance with ASMBS outcome reporting [17]. There were current weight and BMI, percentage of excess weight loss (%EWL), percentage of total weight loss (%TWL) and T2DM changes. T2DM outcomes were defined according to the standardized ASMBS outcome reporting criteria [17]. Complete remission was defined as an HbA1c level < 6.0% and fasting blood glucose < 100 mg/dL without antidiabetic medication, whereas partial remission was established as an HbA1c of 6.0–6.4% and fasting blood glucose of 100–125 mg/dL, also in the absence of glucose-lowering therapy. For the purposes of the present analysis, complete and partial remission were combined into an overall remission category. Improvement was defined as improved glycemic control without achieving remission or a reduction in antidiabetic medication requirements, according to the ASMBS reporting criteria.

All reported results correspond to the specified follow-up period. Remission status was assessed at the last available visit. Surgical techniques and perioperative care protocols—encompassing preoperative, intraoperative, and postoperative interventions—were standardized across all participating centers. At each bariatric unit, patient management was conducted by a comprehensive multidisciplinary team comprising surgeons, physicians, nurses, nutritionists, and psychologists.

### 2.3. Statistical Analysis

Descriptive statistics were utilized to summarize the data. All statistical analyses were performed using Statistica software, version 13.PL (StatSoft Inc., Krakow, Poland). Data normality was assessed via the Shapiro–Wilk test. Because the continuous variables displayed a non-normal distribution, they were expressed as medians with interquartile ranges (IQRs). Group comparisons for continuous data were executed using the Mann–Whitney U test, whereas categorical variables were evaluated using the chi-square test or Fisher’s exact test, as appropriate. A multivariable logistic regression model included clinically relevant variables: sex, age, preoperative BMI, T2DM duration, preoperative insulin treatment, and follow-up duration, regardless of their univariable statistical significance. Postoperative variables were excluded from the regression analysis. T2DM duration was treated as a categorical variable, with <5 years as the reference category. Odds ratios (ORs) with 95% confidence intervals (CIs) were reported. Statistical significance was defined as a *p*-value ≤ 0.05.

### 2.4. Ethical Considerations

The study protocol was strictly reviewed and approved by the Bioethics Committee of the University of Warmia and Mazury in Olsztyn (Approval No. 10/2024) and the Bioethics Committee of the Military Chamber of Physicians in Poland (Approval No. 38/2023). The study was conducted in accordance with the ethical standards of the 1964 Declaration of Helsinki and its subsequent amendments. For both source multicenter retrospective databases (the cohort evaluating outcomes in patients aged >65 years and the longitudinal BARI-10-POL registry), data encryption and complete anonymization protocols were enforced at each participating center prior to central aggregation. In accordance with the national legal framework and institutional guidelines for retrospective non-interventional registry studies using secondary de-identified administrative and clinical data, the requirement for active, explicit patient informed consent was waived by both approving Bioethics Committees.

## 3. Results

### Characteristics

Of the 1703 patients initially assessed, 934 (54.8%) were lost to follow-up, leaving 769 patients with available follow-up data. This proportion refers to the overall source population rather than specifically to older patients with T2DM undergoing SG. Following application of the eligibility criteria, 129 older patients were included in the main analysis (Figure 1).

There were 129 patients analyzed in the study. The median age was 66 years, and 62% were females (Table 1). A total of 37 patients (28.7%) had T2DM for less than 5 years, while 46 (35.7%) for more than 10 years. A total of 24.8% of patients used insulin.

## 4. Outcomes

The cohort was followed for a median duration of 6.0 years (Table 2). The median %TWL was 19.4%, and the median %EWL was 47.8%. A total of 56 (43.4%) patients achieved %EWL > 50%, while 80 (62.0%) patients had remission of T2DM, and 16 (12.4%) patients had no change (Table 2).

There were 8 complications. A total of 8 complications occurred in 8 individual patients (6.2%) within the primary older cohort (N = 129). To standardize reporting, complications were classified using the Clavien–Dindo (CD) grading system:

- Severe Complications (Clavien–Dindo Grade IIIb): Major bleeding, requiring surgical re-operation (n = 4; 3.1%), and sleeve stenoses of the gastric sleeve, necessitating endoscopic or surgical revision (n = 2; 1.6%).
- Moderate Complications (Clavien–Dindo Grade II): Acute rhabdomyolysis, managed successfully with aggressive intravenous fluid resuscitation and medical monitoring (n = 1; 0.8%), and acute superior mesenteric vein thrombosis, requiring systemic anticoagulation and intensive care stabilization (n = 1; 0.8%).

There were no recorded deaths during either the early perioperative phase or the long-term follow-up period within this analyzed sample.

The logistic regression included 110 patients, of whom 68 achieved T2DM remission, and 42 did not. T2DM duration >10 years was associated with lower odds of remission compared with <5 years (OR 0.22, 95% CI 0.07–0.66, *p* = 0.007). Longer follow-up duration was also associated with lower odds of remission (OR 0.86, 95% CI 0.74–0.99, *p* = 0.041) (Table 3).

A comparative analysis was performed with 62 patients aged <60 years who underwent SG for obesity and T2DM. Younger patients achieved greater postoperative weight loss (%TWL 25.5% vs. 19.4%, *p* = 0.020), whereas the T2DM remission rate showed no significant difference between younger and older patients (72.6% vs. 62.0%, *p* = 0.194) (Table 4).

## 5. Discussion

Within this multicenter cohort, T2DM remission was achieved by 62.0% of patients aged 60 years and older following sleeve gastrectomy (SG), while an additional 25.6% experienced improvement. T2DM duration was the main factor associated with remission.

These data underscore that SG is a substantive metabolic intervention for older adults, moving far beyond its traditional role as a weight-reduction surgery. In particular, the ability of SG to restore glucose homeostasis in a considerable proportion of elderly patients highlights its value as a multifaceted therapeutic tool. This is especially relevant in geriatric populations, where conventional pharmacotherapy may be limited by polypharmacy, reduced drug tolerance, and the presence of multiple comorbidities [18,19].

Our data indicate that older patients can derive substantial metabolic benefits from SG. The observed remission rates are consistent with previous reports demonstrating favorable outcomes in older populations, suggesting that chronological age alone should not be considered a contraindication to metabolic surgery [8,9,10,11]. In our cohort, a longer preoperative T2DM duration was significantly associated with lower odds of remission. Specifically, patients with a diabetes history exceeding 10 years had markedly lower odds of achieving remission. This finding aligns with previous studies, suggesting that prolonged hyperglycemia contributes to progressive β-cell dysfunction, potentially reducing the likelihood of postoperative glycemic normalization [15,20,21,22]. These observations highlight the importance of considering diabetes duration when evaluating older patients for metabolic surgery.

In this cohort, commonly reported predictors such as preoperative BMI, age, and insulin use were not statistically significant. Although insulin treatment was associated with lower odds of remission in univariable analysis, this association was not observed after adjustment. These findings should be interpreted cautiously given the limited sample size and potential heterogeneity in baseline diabetes severity.

The weight loss outcomes observed in our cohort are noteworthy, demonstrating a median %EWL of 47.8% and a %TWL of 19.4% at a median follow-up of six years. These findings align with existing long-term studies and support the sustainability of SG in promoting weight reduction, particularly among elderly patients [23,24,25]. Although some studies describe a lower tendency to reduce body weight in older people, in our study, over 43% of patients attained an %EWL exceeding 50%, highlighting the procedure’s efficacy in the demographic aged 60 years and older [23,24,25,26].

In the adjusted analysis, longer preoperative T2DM and follow-up durations were associated with lower odds of remission. Age, sex, preoperative BMI, and insulin treatment were not significantly associated with remission. However, these nonsignificant findings should not be interpreted as evidence of no association, particularly given the limited sample size. Prior studies showed that older patients can achieve comparable metabolic outcomes to younger cohorts [24,25,26,27,28]. The exploratory comparison showed no statistically significant difference in remission between older and younger patients. However, this does not establish equivalence, particularly given the differences in follow-up duration and baseline characteristics.

In our cohort, eight postoperative complications (6.2%) were recorded, with no reported deaths during the available follow-up. This underscores the assertion that SG can be conducted safely among appropriately selected elderly patients. These findings align with prior research suggesting that with meticulous perioperative care and careful patient selection, the associated risks of bariatric surgery in older populations can be effectively mitigated [28,29,30].

Importantly, the additional comparison with patients aged <60 years demonstrated that no significant disparity in T2DM remission rates was observed between younger and older patients, even though the younger cohort achieved significantly higher postoperative weight loss. This indicates that chronological age by itself does not dictate the metabolic outcomes following SG.

When interpreting the role of metabolic bariatric surgery in older populations, it is critical to distinguish formal biochemical diabetes remission from broader, yet equally vital, cardiometabolic benefits. Even in the absence of complete glycemic normalization, older patients frequently experience significant improvements in lipid profiles, arterial hypertension control, and overall cardiovascular risk reduction. This multifaceted metabolic impact is particularly relevant in geriatric care, where conventional pharmacotherapy is often constrained by polypharmacy, poor medication adherence, or compromised drug tolerance [18,19].

Treatment selection in older patients with T2DM should be individualized, considering comorbidities, frailty, and treatment-related risks. Alongside metabolic surgery, contemporary pharmacological therapies, including GLP-1 receptor agonists, dual GIP/GLP-1 receptor agonists, and SGLT2 inhibitors, offer additional metabolic and cardiovascular benefits. Emerging minimally invasive approaches, such as bariatric arterial embolization, are also being investigated [31]. Recent evidence regarding the effects of GLP-1 receptor agonists and SGLT2 inhibitors on atrial fibrillation remains exploratory and requires further investigation [32]. Importantly, our study did not include a nonsurgical comparator, precluding direct conclusions regarding the superiority of any treatment approach.

A key limitation involves the study’s retrospective design, which might have compromised the statistical power required to detect associations for some of the analyzed factors. Larger prospective trials with longer follow-ups are essential to confirm these results and investigate the pathophysiological pathways driving T2DM remission in elderly patients. Moreover, the study did not assess patients’ functional status, frailty, cognitive impairment, polypharmacy or the presence of other comorbidities, which may have influenced the outcomes. The substantial loss to follow-up in the overall source population may have introduced selection and attrition bias. Follow-up completeness, specifically among eligible older patients with T2DM, could not be established, and comparisons with patients lost to follow-up, or sensitivity analyses for missing outcomes, were not feasible.

Another limitation is the lack of complete longitudinal laboratory data, including HbA1c and fasting glucose, as well as more detailed measures of diabetes severity and drugs, which were not uniformly available across participating centers. Furthermore, the three-month medication-free interval required by the 2021 international consensus definition could not be verified, precluding a sensitivity analysis using these criteria. The exploratory comparison between age groups was limited by substantial differences in follow-up duration, incomplete baseline data on T2DM duration, and the inability to adjust for center-related differences. Therefore, these findings should not be interpreted as evidence of equivalent remission rates between age groups.

Our findings support the evidence that MBS plays a significant role in elderly patients diagnosed with T2DM. The data indicate that SG should be regarded as a viable therapeutic alternative for this demographic, especially in cases where the duration of diabetes is relatively brief and traditional medical treatments have not succeeded in attaining sufficient control of the condition.

## 6. Conclusions

SG was associated with substantial T2DM remission at the last available follow-up in patients aged ≥60 years. Longer preoperative T2DM duration was associated with a lower likelihood of remission. Although no statistically significant difference in remission rates was observed between age groups, this exploratory comparison should be interpreted with caution due to differences in follow-up duration and baseline characteristics. Further studies are needed to confirm these findings.

## Figures and Tables

**Figure 1:**
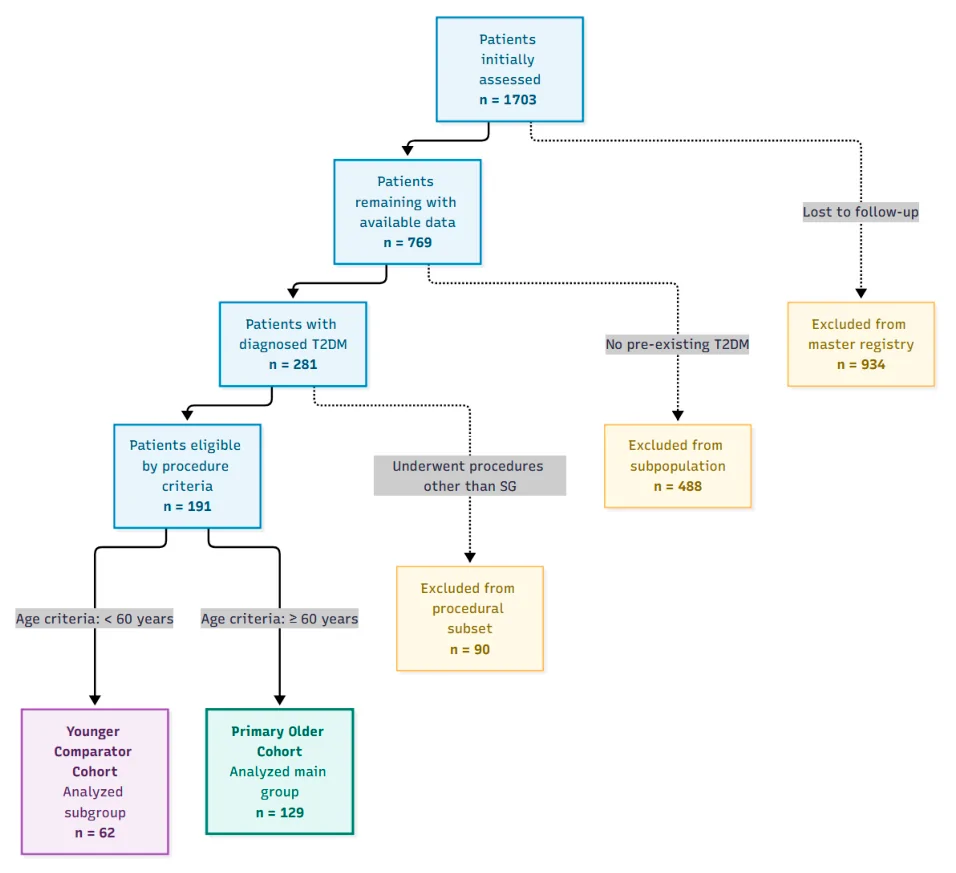
Flow chart of the study.

**Table 1:** Characteristics of patients. BMI: body mass index; HT: hypertension; IQR: interquartile range; T2DM: type 2 diabetes mellitus.

Variable	Value
Age [years], median (IQR)	66.0 (66.0–68.0)
Gender, female (%)	80 (62.0)
Preoperative BMI [kg/m^2^], median (IQR)	42.9 (39.2–46.9)
Length of T2DM	
<5 years, n (%)	37 (28.7)
5–10 years, n (%)	27 (20.9)
>10 years, n (%)	46 (35.7)
Type of treatment of T2DM	
Oral antidiabetic medications, n (%)	97 (75.2)
Insulin, n (%)	32 (24.8)
HT, n (%)	116 (89.9)

**Table 2:** Outcomes of SG.

Variable	Value
Length of stay [days], median (IQR)	2.0 (2.0–3.0)
Operative time [minutes], median (IQR)	61.0 (54.0–90.0)
Complications, n (%)	8 (6.2)
Follow-up [years], median (IQR)	6.0 (4.1–9.5)
Postoperative BMI [kg/m^2^], median (IQR)	33.8 (31.0–38.3)
%EWL, median (IQR)	47.8 (35.0–62.3)
%TWL, median (IQR)	19.4 (14.2–24.8)
T2DM resolution	
Remission, n (%)	80 (62.0)
Improvement, n (%)	33 (25.6)
No changes, n (%)	16 (12.4)

**Table 3:** Univariable and multivariable logistic regression analyses of predictors for T2DM remission.

Variable	Univariable		Multivariable	
Gender, female	1.39 (0.67–2.89)	0.373	1.60 (0.65–3.95)	0.310
Age	0.98 (0.83–1.16)	0.839	1.09 (0.88–1.34)	0.444
Preoperative BMI	0.96 (0.90–1.02)	0.166	0.96 (0.88–1.03)	0.242
Length of T2DM, <5 years, reference				
5–10 years	0.55 (0.18–1.69)	0.298	0.58 (0.16–2.08)	0.405
>10 years	0.23 (0.09–0.61)	0.003	0.22 (0.07–0.66)	0.007
Treatment of T2DM, insulin	0.52 (0.23–1.16)	0.109	1.04 (0.37–2.90)	0.946
HT	1.46 (0.46–4.61)	0.524		
Follow-up duration	0.97 (0.86–1.09)	0.591	0.86 (0.74–0.99)	0.041

**Table 4:** Clinical comparison of younger versus older patients undergoing sleeve gastrectomy.

Variable	<60 Years, n = 62	≥60 Years, n = 129	*p*
Age [years], median (IQR)	44.0 (33.5–50.0)	66.0 (66.0–68.0)	<0.001
Female, n (%)	41 (66.1%)	80 (62.0%)	0.632
Preoperative BMI, median (IQR)	43.7 (39.5–47.5)	42.9 (39.2–46.9)	0.659
**T2DM duration**			**0.056**
<5 years, n (%)	18 (29.0%)	37 (28.7%)	
5–10 years, n (%)	9 (14.5%)	27 (20.9%)	
>10 years, n (%)	7 (11.3%)	46 (35.7%)	
Oral antidiabetic medications, n (%)	37 (59.7%)	97 (75.2%)	0.042
Insulin, n (%)	13 (21.0%)	32 (24.8%)	0.591
Follow-up, years, median (IQR)	10.9 (10.6–12.3)	6.0 (4.1–9.5)	<0.001
Postoperative BMI, median (IQR)	32.3 (28.7–35.4)	33.8 (31.0–38.3)	0.026
%TWL, median (IQR)	25.5 (14.2–33.6)	19.4 (14.2–24.8)	0.020
%EWL, median (IQR)	60.0 (37.1–79.1)	47.8 (35.0–62.3)	0.036
T2DM remission at final visit, n (%)	45 (72.6%)	80 (62.0%)	0.194
Missing duration data, n (%)	**28 (45.2%)**	**19 (14.7%)**	

## Data Availability

The original contributions presented in this study are included in the article. Further inquiries can be directed to the corresponding author.
